# Distinct Roles for CBP and p300 on the RA-Mediated Expression of the Meiosis Commitment Gene Stra8 in Mouse Embryonic Stem Cells

**DOI:** 10.1371/journal.pone.0066076

**Published:** 2013-06-13

**Authors:** Wen Chen, Wenwen Jia, Kai Wang, Xiaoxing Si, Songcheng Zhu, Tao Duan, Jiuhong Kang

**Affiliations:** 1 Clinical and Translational Research Center of Shanghai First Maternity and Infant Health Hospital, Shanghai Key Laboratory of Signaling and Disease Research, School of Life Science and Technology, Tongji University, Shanghai, People’s Republic of China; 2 Department of Obstetrics, Shanghai First Maternity and Infant Hospital, Tongji University School of Medicine, Shanghai, People’s Republic of China; University of Kansas Medical Center, United States of America

## Abstract

In mammalian germ cells, meiotic commitment requires the expression of Stimulated by retinoic acid gene 8 (Stra8), which is transcriptionally activated by retinoic acid (RA). However, little is known about the epigenetic mechanism by which RA induces Stra8 expression. Utilizing a chromatin immunoprecipitation assay (ChIP), we showed that RA increases histone acetylation at the Stra8 promoter in murine embryonic stem cells (ESCs), a model for germ cell differentiation. Furthermore, we explored whether two coregulators with histone acetyltransferase (HAT) activity, Creb-binding protein (CBP) and p300, are involved in the activation of Stra8. The lentiviral shRNA knockdown of endogenous CBP led to Stra8 repression, while the overexpression of CBP enhanced Stra8 expression at both the mRNA and protein levels. ChIP analysis confirmed that CBP is the crucial coactivator for RA-mediated Stra8 transcription and that it enhances the level of histone acetylation and recruits RNA polymerase II to establish transcriptionally active chromatin. Furthermore, shRNA of p300 enhanced Stra8 expression, and the overexpression of p300 reduced Stra8 expression, independently of its HAT activity. ChIP showed that the knockdown of p300 significantly increased the level of CBP at the Stra8 promoter. These findings demonstrate that CBP and p300 play distinct roles in RA-mediated Stra8 gene transcription.

## Introduction

RA, an active metabolite of vitamin A, modulates various events in cellular proliferation, differentiation, and development [1], [2]. In particular, the addition of RA to the culture medium could establish and enhance the microenvironment that ESCs rely upon for differentiation into germ cells [3]–[5]. RA induces differentiation primarily by binding to specific nuclear hormone receptors (retinoic acid receptors, or RARs), which form an obligatory heterodimer with their paralogs, retinoid X receptors (RXRs). These heterodimers bind RAREs (retinoic acid responsive elements) in target genes in the nucleus [6], [7]. RAR-RXRs contribute to the dynamic remodeling of local chromatin structure at the level of target genes containing RAREs by recruiting coregulator complexes with histone acetyltransferase (HAT) or histone deacetylase (HDAC) activity, respectively, and thereby activate or repress gene expression [8], [9].

Numerous studies have shown RA is the key molecular switch that underpins the sex-specific timing of meiotic entry in mammalian embryonic gonads, although RA may not be the only inducer that controls Stra8 expression in the meiotic initiation [10]. The onset of meiosis occurs earlier in the ovary (E13.5) than in the testis (after birth) [11], [12]. Despite the different timing of the meiotic entry, male and female germ cells may share an identical meiotic initiation pathway, in which RA induces Stra8 gene expression in premeiotic germ cells. Gene knockout studies have demonstrated that Stra8 is required for meiotic initiation and meiotic progression in germ cells of both sexes [13]. Although most of these studies reinforce the importance of RA and Stra8 in gametogenesis, it remains unclear how RA regulates Stra8 expression. Our previous studies showed that RA indirectly enhances the expression of Stra8 and other germ cell genes through the Smad pathway [14]. Because the Stra8 promoter has two putative RA-response element sequences [15], RA can also act directly on the Stra8 gene. Recent studies in F9 premeiotic germ cells have shown that RA-induced Stra8 transcription is epigenetically repressed by HDACs [16]. However, the precise mechanism of histone acetylation in RA-mediated Stra8 expression is also currently unclear.

CBP and p300, which possess intrinsic HAT activity and form the two-member KAT3 family of HATs, are known coregulators of nuclear hormone receptors. These proteins can enhance transcriptional activity either through their protein acetyltransferase activity or by acting as scaffold proteins to recruit other coregulators or components of the basal transcription machinery [8], [17]–[19]. Moreover, sumoylated p300 was shown to repress gene expression [20]. The high degree of homology between CBP and p300 suggests that these proteins could, at least in part, be functionally redundant. Indeed, it has been shown that CBP and p300 perform some redundant functions. However, the phenotypic changes observed in knock-out mice indicate that CBP and p300 have unique functions [21], [22]. Although in vitro studies have demonstrated similar functions for CBP and p300 in most cases, accumulating evidence has suggested that they have different functions in vivo and that the expression of a specific gene may preferentially require the activity of one protein rather than the other [23]–[27]. In this study, we explored the individual contributions that CBP and p300 make to RA-regulated Stra8 gene expression in the ESCs, a model for germ cell differentiation. Our studies demonstrated that CBP serves as a coactivator in RA-induced Stra8 transcription, while p300 represses Stra8 expression through a mechanism independent of its HAT activity.

## Results

### RA Increased Histone Acetylation at the Stra8 Promoter

**Figure 1:**
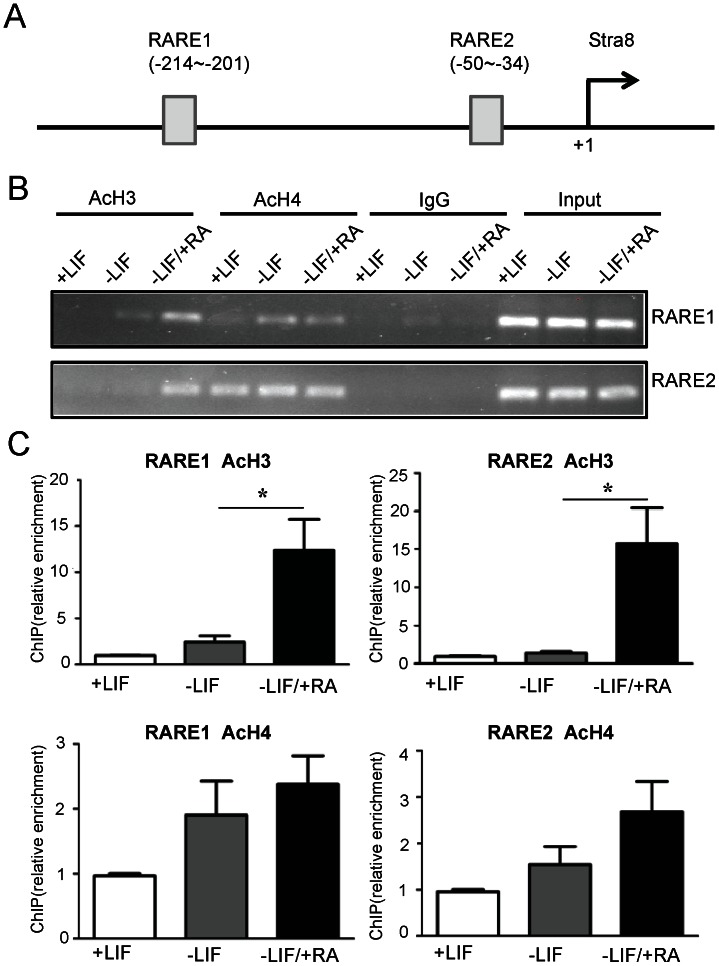
Histone acetylation levels at the Stra8 promoter during the RA-induced differentiation of mouse ESCs. (**A**) Diagram of the promoter of the Stra8 gene, measured in bp. (**B**) ChIP analysis of AcH3 and AcH4 occupancy on the RARE1 and RARE2 regions of the Stra8 promoter in ESCs treated with +LIF, −LIF or −LIF/+RA- for 24 h. Isolated chromatin was immunoprecipitated with a nonspecific rabbit normal IgG as a control. The input represents 10% of the material used for immunoprecipitation. (**C**) The AcH3 and AcH4 levels at the Stra8 promoter, as determined by ChIP analysis in ESCs treated with +LIF, −LIF or −LIF/+RA- for 24 h. Data are means ± SEM (n = 3). *P*<0.05(*) vs. −LIF ESCs (One-way ANOVA with Turkey-Kramer post hoc test).

The acetylation of core histone proteins is usually interpreted as a marker of actively transcribed genes. To test whether the acetylation status of histone proteins on Stra8 gene promoter changes under RA treatment, we used a ChIP assay to measure the levels of acetylation of histone H3 (AcH3) and histone H4 (AcH4) in the Stra8 promoter. Stra8 has two putative RAREs, all located upstream of the transcription start site (Fig. 1A). For our ChIP studies, we examined the RARE1 and RARE2 regions. Comparing with +LIF control, the withdrawal of LIF (−LIF) did not significantly increase the levels of AcH3 and AcH4 at both RARE1 and RARE2 regions. In contrast, by comparing ESCs treated with 1 µM RA for 24 h (−LIF/+RA) with non-LIF-treated cells (−LIF), we observed that RA significantly increases the AcH3 level at both RARE1 and RARE2 in the Stra8 gene, but does not increase the AcH4 level at the two RAREs regions. (Fig. 1, B and C). These results indicated that RA induces high histone acetylation at the Stra8 promoter.

### CBP and p300 Differentially Regulate RA-induced Stra8 Gene Expression

**Figure 2:**
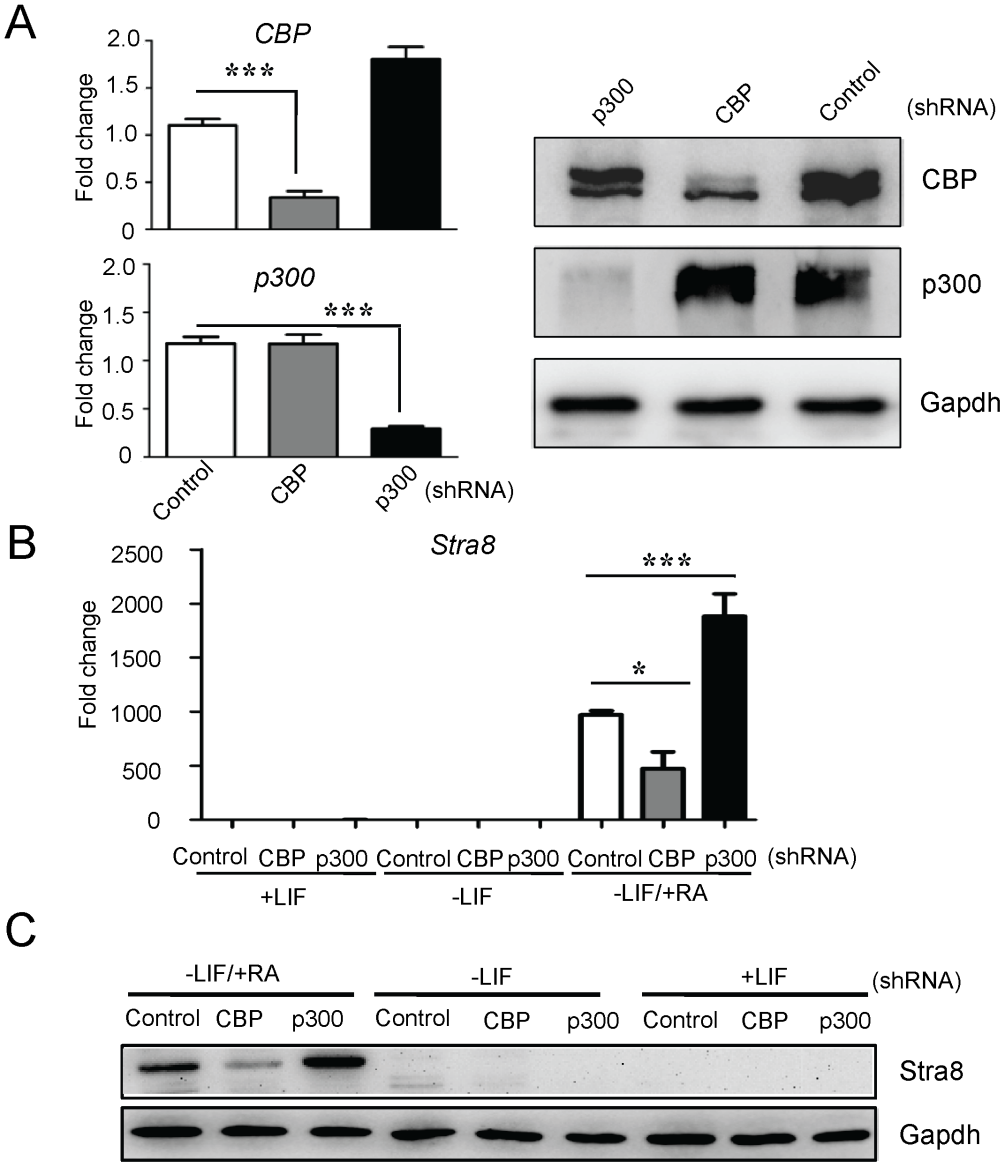
The effects of CBP and p300 knockdown on RA-mediated Stra8 expression in mouse ESCs. (**A**) Real-time PCR and western blot analysis to measure the mRNA and protein levels of CBP and p300 in control, CBP or p300 shRNA-infected ESCs. The mRNA expression levels were normalized to *Gapdh* and presented as fold change over control set. Data are means ± SEM (n = 3). *P*<0.001(***) vs. control shRNA cultures (One-way ANOVA with Turkey-Kramer post hoc test). Gapdh was used as a protein loading control. (**B**) Real-time PCR to analyze the Stra8 expression in control, CBP or p300 shRNA-infected ESCs after treatment with +LIF, −LIF or −LIF/+RA for 24 h, as indicated. The mRNA expression levels were normalized to *Gapdh* and presented as fold change over control shRNA (+LIF) set. Data are means ±SEM (n = 3). *P*<0.05(*) and *P*<0.001(***) vs. control shRNA cultures under RA treatment (One-way ANOVA with Turkey-Kramer post hoc test). (**C**) Western blot to analyze the protein levels of Stra8 in control, CBP or p300 shRNA-infected ESCs following treatment with +LIF, −LIF or −LIF/+RA for 24 h. Gapdh was used as a protein loading control.

To test whether two HATs, CBP and its paralog p300, are involved in Stra8 gene activation, we knocked down CBP or p300 expression with shRNA lentiviruses. The cellular mRNA levels of CBP and p300 were very significantly decreased in ESCs infected with lentiviruses expressing CBP- and p300-targeted shRNAs, respectively, compared to ESCs infected with control lentiviruses. The protein levels of CBP and p300 protein levels were examined with Western blot analysis, which also demonstrated dramatic knockdown and confirmed the specificity of the respective shRNAs and antibodies (Fig. 2A). As shown in Fig. 2 B and C, only withdrawal of LIF did not increase the Stra8 expression. The knockdown of CBP decreased the RA-induced levels of Stra8 mRNA and protein, whereas p300 depletion increased Stra8 expression. These results demonstrated that CBP and p300 regulate RA-induced Stra8 expression in different ways.

### The HAT Activity of CBP, but not p300, is Important for Stra8 Activation

**Figure 3:**
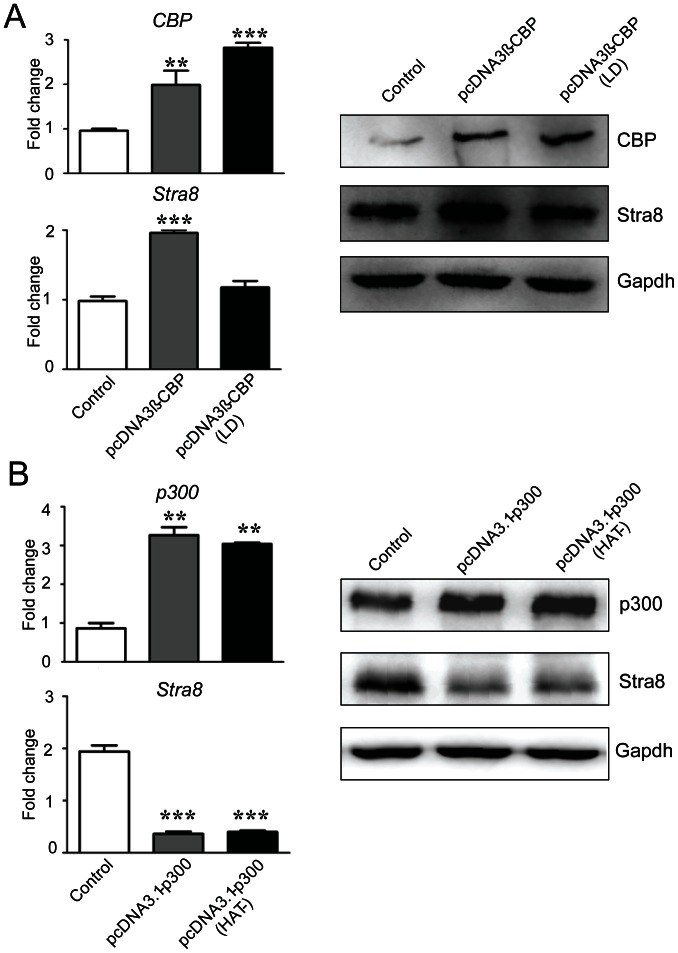
The activation of the Stra8 gene by CBP requires its HAT activity, while p300 represses Stra8 expression through a HAT-independent mechanism. (**A**) Real-time PCR and Western blot analysis of the expression of Stra8 in ESCs after transfection with pcDNA3ß-CBP or pcDNA3ß-CBP (LD) and treatment with RA for 24 h. (**B**) Real-time PCR and WB to analyze the Stra8 expression in ESCs after transfection with pcDNA3.1-p300 or pcDNA3.1-p300 (HAT-) and treatment with RA for 24 h. In Real-time PCR analysis of Figure 3, the mRNA expression levels were normalized to *Gapdh* and presented as fold change over control set. Data are means ± SEM (n = 3). *P*<0.01(**) and *P*<0.001(***) vs. control ESCs (One-way ANOVA with Turkey-Kramer post hoc test).

To better characterize the roles of CBP and p300 in Stra8 regulation and further explore the potential involvement of CBP/p300 HAT activity in the acetylation of the Stra8 promoter, we compared the regulation of RA-induced Stra8 expression by wild type CBP and p300 and mutant CBP and p300 that lack functional HAT domains. As shown in Fig. 3A, wild-type CBP can increase RA-mediated Stra8 expression at both the mRNA and protein levels, while an enzymatically deficient mutant of CBP (CBP-LD) fails to do so. In contrast, both the wild-type and (HAT-) mutant p300 dramatically decreased the expression levels of Stra8 (Fig. 3B). This further demonstrated that CBP and p300 play distinct roles in RA-induced Stra8 expression. Furthermore, these results showed that the activation of Stra8 gene expression by CBP requires its HAT activity.

### RA Increased the Recruitment of CBP to Establish Transcriptionally Active Chromatin on the Stra8 Promoter

**Figure 4:**
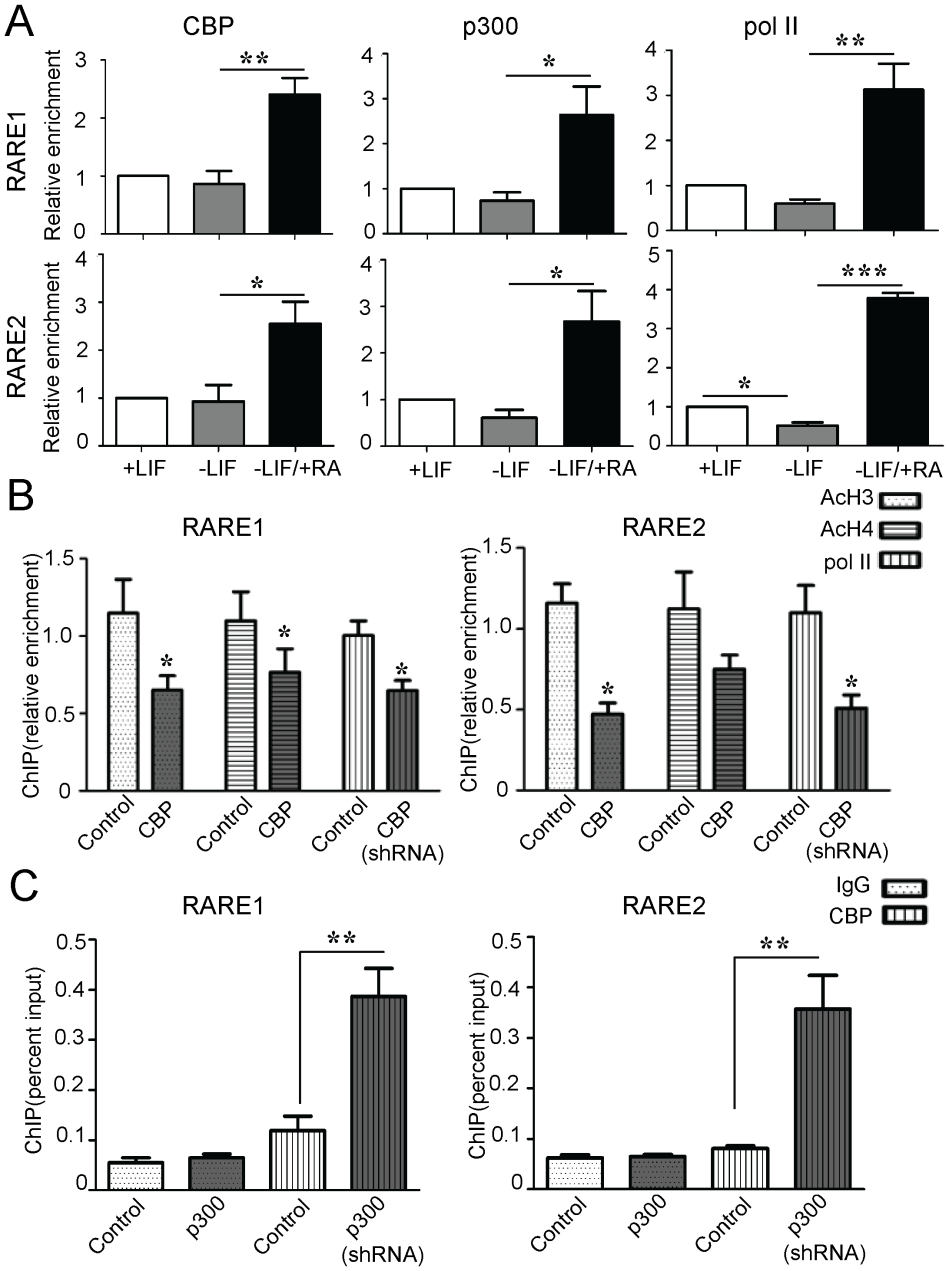
RA increases CBP occupancy at the Stra8 promoter with concomitant increase in histone acetylation and RNA polymerase II recruitment. (**A**) ChIP analysis of p300, CBP and Pol II (RNA polymerase II) occupancy of the Stra8 promoter in ESCs treated with +LIF, −LIF or −LIF/+RA for 2 h. Data are means ± SEM (n = 3). *P*<0.05(*), *P*<0.01(**) and *P*<0.001(***) vs. −LIF ESCs (One-way ANOVA with Turkey-Kramer post hoc test). (**B**) ChIP analysis was performed in control and CBP shRNA-infected ESCs treated with RA for 24 h. *P*<0.05(*) vs. control shRNA cultures (Student’s *t*-test). Data are means ± SEM (n = 3). (**C**) ChIP analysis of CBP occupancy of the Stra8 promoter in control and p300 shRNA-infected ESCs following treatment with −LIF/+RA for 1 h. Data are means ± SEM (n = 3). *P*<0.05(*) vs. control shRNA-infected ESCs using the CBP antibody (Student’s *t*-test).

To confirm that CBP is crucial for RA-mediated Stra8 transcription, we used a ChIP assay to examine the endogenous occupancy of CBP, p300 and RNA polymerase II at the Stra8 promoter in the presence and absence of RA. As shown in Fig. 4A, comparing with LIF-treated controls, the withdrawal of LIF did not induce the recruitment of CBP, p300 or RNA polymerase II on the both the RARE1 and RARE2 regions. In contrast, RA increased significantly the levels of CBP, p300 and RNA polymerase II at RAREs of the Stra8 gene at 2 hours of treatment.

Having defined RA-induced histone acetylation and RNA polymerase II recruitment at the promoter of Stra8 gene, we examined the effects of CBP knockdown to determine the position of CBP in the sequence of events leading to transcriptional activation. CBP knockdown significantly reduced the RA-induced increases in AcH3 acetylation and RNA polymerase II occupancy at both the RARE1 and RARE2 regions, and it also decreased the AcH4 level at the RARE1 region (Fig. 4B). Moreover, lentiviral-mediated knockdown of p300 triggered a robust increase in CBP occupancy of both the RARE1 and RARE2 regions (Fig. 4C). Thus, CBP is involved in acetylating histones at the Stra8 gene promoter, promoting an open chromatin structure that facilitates RNA polymerase II recruitment.

## Discussion

Despite the essential roles of RA and Stra8 in mouse germ cell development, it is not clear how RA activates Stra8 expression, especially at the epigenetic level. Using mouse ESCs, a model for germ cell differentiation, we demonstrated that RA increases histone acetylation at the Stra8 promoter. Further, we found that the histone acetylases CBP and p300 play distinct roles in Stra8 gene transcription. RA-signaling recruits CBP to the Stra8 promoter, which in turn enhances the level of AcH3 and AcH4, recruits RNA polymerase II to establish transcriptionally active chromatin, and activates Stra8 expression. In contrast, the presence of p300 leads to the repression of Stra8.

Certain histone modifications, particularly the acetylation of histone N-terminal tails, are correlated with the activation of gene transcription. A recent study reported that oogenesis can be induced in adult female mice by the HDAC inhibitor TSA, indicating that histone acetylation status may dictate whether germ cells can enter meiosis [28]. In the current study, greater than 10-fold increases in the AcH3 levels at the Stra8 promoter were observed upon RA treatment, and these changes did not occur during LIF withdrawal alone (Fig. 1). In a previous study, we found that taking away LIF does not change Stra8 promoter activity in mouse ESCs compared with LIF treatment [14]. Consist with it, we observed that Stra8 expression peaked at approximately 1000-fold higher than basal levels 24 h after RA treatment, but there was no significant change in Stra8 expression in ESCs when only LIF was withdrawn. These data suggest that this increase in expression is associated with increases in histone acetylation. Furthermore, ChIP analysis indicated that CBP is recruited to the Stra8 promoter in response to RA (Fig. 4A), and the decreases in AcH3 and AcH4 levels at the Stra8 promoter after CBP knockdown demonstrate that CBP is related to the histone acetylation within the Stra8 promoter (Fig. 4B). Consistent with this model, the activation of Stra8 expression by CBP is dependent on its HAT activity, as shown by comparing the effects of wild-type CBP and its HAT mutant, CBP-LD (Fig. 3A). The level of histone acetylation is regulated by HATs and HDACs. In previous studies, the activation of the Stra8 promoter in premeiotic germ cells was repressed by HDACs, and the HDAC inhibitor TSA can maximize RA-induced Stra8 transcription [16]. Gene chip analysis in F9 embryonic carcinoma cells revealed that Stra8 can be positively regulated by either RA or TSA [29]. Thus, a functional interaction between the protein acetylase CBP and a TSA-sensitive pathway may be responsible for the bulk of RA-induced Stra8 gene expression. Other investigators have also observed that the CBP and deacetylases together mediate HIF-responsive gene transcription [30].

Accumulating evidence in cell-based studies indicates that CBP and p300 are not completely redundant but also have unique roles. A genome-wide screen using ChIP-seq on T98G glioblastoma cells identified significant differences in the binding levels and targets of p300 and CBP [25]. In a microarray experiment, p300 was found to be dominant over CBP in advanced prostate cancer cells [24]. In another study, survivin gene expression was activated by enhancing the interaction of β-catenin with CBP, while interaction with p300 repressed its expression [23]. Using lentiviral shRNA and ChIP analysis, our study presents strong evidence that CBP and p300 have distinct functions in the regulation of RA-mediated Stra8 transcription in mouse ESCs. The shRNA depletion of endogenous CBP led to transcriptional repression, while the overexpression of CBP enhanced Stra8 expression at both the mRNA and protein levels (Fig. 2 and 3A). ChIP analysis of the Stra8 gene in RA-treated cells after CBP depletion showed that CBP is required for the high histone acetylation and occupancy of RNA polymerase II at the Stra8 promoter (Fig. 4B). These results demonstrate that CBP may serve as a coactivator in RA-induced Stra8 gene transcription. In contrast, the knockdown of p300 enhances RA-mediated Stra8 expression, and the overexpression of p300 reduces Stra8 expression through a mechanism independent of its HAT domain, suggesting that p300-mediated Stra8 repression may not require its HAT activity (Fig. 2 and 3B). The ChIP results also showed that the knockdown of p300 significantly increased the level of CBP at the Stra8 promoter (Fig. 4C). Previous studies concluded that either CBP or p300 can bind RA-bound RARs via the AF2 domains of the RARs [18], [31], suggesting that these proteins may compete for the RARs within the Stra8 promoter. A role for p300 as a corepressor of gene transcription would not be unprecedented [20], [23], [32]. Although the mechanism by which p300 may act to repress Stra8 gene transcription is still unclear, a recent report suggests that the SUMO-1 modification of p300 results in transcriptional suppression [20]. An indispensable role of the SUMO-1 pathway in mammalian meiosis has also been reported recently [33]. Further studies to explore how p300 represses RA-induced Stra8 expression and whether this activity is associated with SUMO-1 modification are in progress.

Based on our results, we propose a model of the events occurring during the RA-regulation of the Stra8 gene in a CBP-dependent fashion. The RA-induced recruitment of CBP leads to increases in AcH3 and AcH4 at the Stra8 promoter. RNA polymerase II is recruited to the promoter, and transcription ensues. However, when CBP is depleted, RA fails to induce histone acetylation and the recruitment of RNA polymerase II, inhibiting RA-induced Stra8 expression. Overall, the results of this study show that RA induces histone acetylation at the Stra8 promoter. We further show that the HAT enzyme CBP plays positive roles in RA-mediated chromatin remodeling of the Stra8 gene, whereas the related protein p300 represses Stra8 expression through a HAT-independent activity. Given the wide use of RA and the Stra8 gene as a pre-meiosis marker in different germ cell differentiation strategies [3]–[5], understanding the mechanism through which Stra8 activity is regulated by RA has important implications for the derivation of gametes from ESCs in vitro.

## Materials and Methods

### Mouse ESC Culture

Commercially available mESCs (E14.1) were provided by the Cell Bank of Shanghai Institute for Biological Sciences, Chinese Academy of Sciences [34]. Cells were cultured on plates coated with 0.1% gelatin in high glucose Dulbecco’s modified Eagle’s medium (DMEM; Gibco) supplemented with 15% ES cell qualified fetal bovine serum (Gibco), 1% penicillin and streptomycin (Gibco), 2 mM L-glutamine (Gibco), 0.1 mM non-essential amino acid (NEAA; Gibco), 0.1 mM 2-mercaptoethanol (Gibco) and 1000 units/ml of LIF (Chemicon). For differentiation, cells were maintained in culture media without LIF, and 1 µM RA (Sigma) was added.

### Knockdown of CBP or p300 in ESCs

Short hairpin RNA (shRNA) nucleotides corresponding to the gene under investigation were cloned into the *Age*I and *Eco*RI sites of the pLKO.1 RNAi plasmid. The shRNA oligonucleotides used were as follows: CBP RNAi: 5′-TAA CTC TGG CCA TAG CTT AAT-3′, p300 RNAi: 5′-CCC TGG ATT AAG TTT GAT AAA-3′. To generate knockdown cells, ESCs were infected separately with the shRNA lentivirus, and 1 µg/ml puromycin was added 48 hours after transfection [14]. Real-time PCR and western blot were also used to determine the effectiveness of the CBP and p300 knockdowns.

### Transient Plasmid Transfection

1×10^5^ suspended ESCs per well in 6-well plates were transfected with plasmids using the transfection reagent GeneExpresso Max (Excellgen) in LIF medium, according to the manufacturer’s instructions. At 24 h post-transfection, the cells were treated with 1 µM RA for 24 h. For CBP overexpression, 0.1 µg pcDNA3β-FLAG-CBP-HA (Addgene, 32908) or pcDNA3β-FLAG-CBP-LD-HA (HAT enzyme-dead mutant: L1435A/D1436A, Addgene, 32906) plasmids were used. For p300 overexpression, 1.0 µg pcDNA3.1-p300 (Addgene, 23252) or pcDNA3.1-p300 (HAT-) (HAT enzyme-dead mutant: H1415A/E1423A/Y1424A/L1428S/Y1430A/H1434A, Addgene, 23254) plasmids were used.

### Reverse Transcription and Real-time PCR Analysis

Total RNA was isolated using the TRIzol reagent (Invitrogen) following the manufacturer’s instructions. One microgram of RNA from each sample was reverse-transcribed to cDNA using PrimeScript™ RT reagent kit (TaKaRa). cDNA was amplified in a 20 µl reaction with the Takara Ex Taq PCR kit (TaKaRa). PCR amplification was conducted on an automated Stratagene Mx3000 QPCR system (Stratagene). Relative gene expression data were analyzed with the 2^−ΔΔ^CT method. The specificity of the PCR was verified by both melting curve and gel analysis. The following PCR primers were used: *CBP* forward 5′-GAC CGC TTT GTT TAT ACC TGC-3′ and reverse 5′-TCT TAT GGG TGT GGC TCT TTG -3′; *P300* forward 5′-GTT GCT ATG GGA AAC AGT TAT GC -3′ and reverse 5′-TGT AGT TTG AGG TTG GGA AGG -3′; *Stra8* forward 5′- GAG GCC CAG CAT ATG TCT AAC-3′ and reverse 5′-GCT CTG GTT CCT GGT TTA ATG-3′; *Gapdh* forward 5′-AGG TCG GTG TGA ACG GAT TTG-3′ and reverse 5′-TGT AGA CCA TGT AGT TGA GGT CA-3′.

### Western Blot Analysis

Cultured cells were washed twice with cold PBS, harvested, and lysed in RIPA buffer (1% Triton X-100, 0.5% sodium deoxycholate, 0.1 mM PMSF, 0.1% sodium dodecyl sulfate (SDS), 150 mM NaCl, 50 mM Tris (pH 7.4) with Halt™ Protease Inhibitor Cocktails (Thermo)). The lysates were centrifuged and protein concentrations of the supernatant were estimated using the BCA protein assay kit (Pierce). Protein samples (15 µg) were separated on 10% SDS-PAGE gels and electroblotted onto PVDF membranes (Millipore). Primary antibodies for CBP (A-22, 1∶1000, Santa Cruz Biotechnology), p300 (C-20, 1∶1000, Santa Cruz Biotechnology), Stra8 (ab49602, 1∶1000, Abcam), AcH4 (06-866, 1∶1000, Millipore), AcH3 (06-599, 1∶3000, Millipore) and glyceraldehyde-3-phosphate dehydrogenase (GAPDH, 1∶1000, Santa Cruz Biotechnology) were incubated overnight at 4°C, followed by incubation with the appropriate HRP (horseradish peroxidase)-conjugated secondary antibodies. HRP was detected using the SuperSignal West Pico Chemiluminescent Substrate (Pierce).

### ChIP Assay

Cells (6×10^6^) were crosslinked with 1% formaldehyde for 10 min, quenched with 125 mM glycine for 5 min, and sonicated for 50 s (in repeated cycles of 10 s on and 20 s off) using a Bioruptor sonicator (Diagenode) kept at 4°C to achieve fragments that were 200–1000 bp in size. The samples were precleared with protein G-agarose/salmon sperm DNA (Millipore) for 1 h at 4°C followed by an overnight incubation at 4°C with the following antibodies: 5 µg of CBP (A-22, Santa Cruz Biotechnology), 5 µg of p300 (C-20, Santa Cruz Biotechnology), 2 µg RNA polymerase II clone CTD4H8 (Millipore), 10 µg of AcH4 (06-866, Millipore), 10 µg of AcH3 (06-599, Millipore) and 1 µg of normal rabbit/mouse IgG (Millipore). The immune complexes were precipitated with 60 µl G-agarose for 1 h. Then, the immunoprecipitates were washed and eluted, and purified DNA was obtained. Ten percent of the total genomic DNA from the nuclear extract was used as the input. Purified immunoprecipitated DNA and input DNA were used as a template for subsequent real-time PCR. ChIP primer sets were checked for linear amplification and designed to amplify two separate regions of the mouse Stra8 promoter [16]: RARE1 forward (5′-TGG CAT TGC CCT GGT TGA GGG G-3′) and reverse (5′-CAA CTT GCC ACA GGT TGC AAG AGG-3′), spanning nucleotides −265 to −160; RARE2 forward (5′-GTG ACA GGG CTG TGA TTG GTT CGC-3′) and reverse (5′-CTC ACG ACT GCC CGT CGC AG-3′), spanning nucleotides −87 to +11.

### Data Analysis

GraphPad Prism 4.0 (GraphPad Software) was used for data analysis, and the data were calculated as the means ± SEM for three independent experiments. Comparisons between means were performed by one-way analysis of variance (ANOVA) with the Turkey-Kramer post hoc test for multiple (>2) groups or Student’s *t*-test for comparing two means of independent samples. Differences were considered to be significant at *P*<0.05(*), *P*<0.01(**) and *P*<0.001(***).
